# Review: Adolescents' perspectives on and experiences with post‐primary school‐based suicide prevention as end‐users, co‐creators and peer helpers – a systematic review meta‐ethnography

**DOI:** 10.1111/camh.70043

**Published:** 2025-11-06

**Authors:** Eibhlin H. Walsh, Laura Hemming, Matthew P. Herring, Jennifer McMahon

**Affiliations:** ^1^ School, Child & Youth (SCY) Mental Health and Wellbeing Research Lab, Health Research Institute University of Limerick Limerick Ireland; ^2^ Department of Psychology University of Limerick Limerick Ireland; ^3^ Violet Vines Marshman Centre, La Trobe Rural Health School Melbourne Vic. Australia; ^4^ Physical Activity for Health Cluster, Health Research Institute University of Limerick Limerick Ireland; ^5^ Department of Physical Education and Sports Sciences University of Limerick Limerick Ireland

**Keywords:** Adolescence, suicide, prevention, school, qualitative methods

## Abstract

**Background:**

Suicide is the fourth leading cause of death in adolescents globally. Post‐primary school‐based suicide prevention (PSSP) interventions are an evidence‐based suicide prevention strategy. However, adolescents' experiences with PSSP interventions are not well understood but are arguably critical to ensuring PSSP interventions have their intended impacts on adolescent mental health. No known review synthesising adolescents' PSSP intervention experiences exists. The objective of the meta‐ethnography review is to explore the perspectives and experiences of adolescents engaging with PSSP interventions, as participants/end‐users, intervention advisors, facilitators, co‐designers and co‐researchers.

**Method:**

A peer‐reviewed meta‐ethnography protocol has been published. The meta‐ethnographic approach followed Noblit and Hare's seven‐stage process for conducting meta‐ethnography and adhered to the eMERGe reporting and the PRISMA statement guidelines. Searches of nine databases identified journal articles and dissertations. Study quality appraisal was undertaken in duplicate using the CASP checklist.

**Results:**

Sixteen journal articles and dissertations were retained for analysis. Adolescents were engaged in PSSP interventions as end‐users, co‐creators and peer helpers. Reciprocal translations and lines of argument (LOA) synthesis reveal the importance of the following aspects of engaging with PSSP interventions: (1) *how* adolescents were engaged; (2) trust in facilitators, peers, school personnel and schools; and (3) personal experience sharing for connection and engagement. Our findings demonstrate the intersection between school contexts and adolescents' experiences with PSSP interventions.

**Conclusion:**

To our knowledge, this is the first review to synthesise qualitative findings of adolescents' experiences with engaging with PSSP interventions. Understanding and harnessing adolescents' perspectives is key for enhancing PSSP intervention effectiveness and implementation. Our findings are relevant to broader health‐related fields and practice, particularly given the increasing calls for youth voice.

**Key Practitioner Message**

**What is known?**

- PSSP interventions are an evidence‐based suicide prevention strategy and understanding adolescents' experiences is important for interventions to have their intended impacts on addressing adolescent suicidal behaviour
- Understanding adolescents' perspectives on interventions concerning them aligns with Article 12 of the United Nations' Convention on the Rights of the Child, which mandates that young people have a right to express their views on matters concerning them. However, no known review of adolescents experiences of engaging with PSSP intervention exists

**What is new?**

- This meta‐ethnography review explores adolescents' experiences of engaging with PSSP interventions by identifying and collating qualitative findings from 16 studies. Findings show that adolescents enjoyed active engagement and personal experience sharing as intervention components, and particularly valued trust in intervention facilitators, peers, school personnel and schools
- Notably, aspects of school contexts, including relationships and culture around mental health, have the potential to both undermine and amplify how adolescents engage with interventions

**What is the significance of this for clinical practice?**

- The following considerations are important for how adolescents engage with PSSP interventions in school settings: (1) adolescent voice in PSSP interventions; (2) peer‐ and school personnel relationships; and (3) schools' culture and attitudes towards mental health and suicide
- In practice, options to engage as part of smaller groups and opt‐out intervention activities can be useful for enhancing adolescents' intervention engagement

## Introduction

Globally, suicide is the fourth leading cause of death in 15‐ to 19‐year‐olds (World Health Organisation [WHO], 2021b). Suicidal thoughts and behaviours (STBs) include suicide attempts (SA) (i.e. non‐fatal injurious behaviour to oneself with intent of death) and suicidal ideation (SI) (i.e. thinking about or considering suicide) (Hughes et al., 2023). There are far‐reaching societal impacts of adolescent suicide, with exposure to peer suicide in adolescence predicting SA in adulthood (Geoffroy, Orri, Girard, Perret, & Turecki, 2021). Global calls recommend situating approaches to suicide prevention in settings which serve adolescents, including schools (WHO, 2021a). Post‐primary, school‐based suicide prevention (PSSP) interventions are an evidence‐based strategy showing effectiveness in reducing adolescent STBs (Robinson et al., 2018; Walsh, McMahon, & Herring, 2022). Levels of PSSP preventive strategies include the following: (1) universal (i.e. school/classroom interventions for the wider population), (2) selective (i.e. identification of at‐risk adolescents) and (3) indicated (i.e. targeting at‐risk adolescents' STBs) (Goldsmith, 2002). PSSP interventions can also incorporate upstream strategies targeting STBs as secondary intervention outcomes alongside other health and well‐being outcomes (Walsh, McMahon, & Herring, 2022).

PSSP intervention impacts vary considerably across schooling contexts, which are multi‐faceted, complex and dynamic environments (Joyce & Cartwright, 2020), for instance, classrooms comprise teachers–student interactions that are nested in schools characterised by unique cultures and practices, which collectively are located in wider diverse communities (Domitrovich et al., 2008; Hoagwood & Johnson, 2003). However, consideration of macro‐, school‐ and individual‐level contextual factors is lacking in the extant PSSP literature, with investigation of factors relating to adolescents themselves particularly scant (Walsh, Herring, & McMahon, 2022), despite arguably being among the most important with respect to PSSP intervention effectiveness and implementation. Adolescents' perceptions of school‐based mental health interventions were among the most critical for interventions to achieve their intended mental health outcomes, in comparison with typical outcome indicators, such as intervention quality and model (Rojas‐Andrade & Bahamondes, 2019). As such, understanding how adolescents experience interventions should be a priority gap to address in the PSSP field.

Youth voice represents young people's viewpoints and involvement in services, organisations, schools and government (Garvey, McIntyre‐Craig, & Myers, 2000, as cited in Havlicek, Lin, & Braun, 2016). Intervention engagement represents how individuals are involved in interventions in both research and practice, including participation as end‐users to planning, design, analysis, translation and dissemination (Campus Engage, 2017; Dunne & Owen, 2013). We have adapted learning definitions to define how adolescents engage with PSSP interventions; didactic engagement positions adolescents as passive knowledge receivers (i.e. teacher instruction); experiential engagement fosters self‐directed discovery (Hovelynck, 2002) (i.e. group or reflective activities); and agentic engagement involves youth‐initiated collaborative and goal‐congruent action (Reeve, 2013) (i.e. intervention co‐design).

The importance of giving due consideration to adolescents' experiences with PSSP interventions is underscored by Article 12 of the United Nation Convention on the Rights of the Child, which mandates that young people have the right to voice their views on matters concerning them (United Nations Human Rights, 1990). Qualitative research with adolescents enhances our knowledge on adolescents' unique and nuanced perspectives and experiences (Wasserman et al., 2018). Systematic reviews can broaden and contextualise the understanding of qualitative evidence by drawing research from varying contexts and methodologies together to generate insights pertinent to intervention effectiveness and implementation useful for researchers, practitioners and decision‐makers (Campbell et al., 2011; Lorenc, Pearson, Jamal, Cooper, & Garside, 2012). Meta‐ethnography is an interpretative qualitative synthesis method which can generate insights that go beyond the individual study findings (Campbell et al., 2011; Mays, Pope, & Popay, 2005; Noblit & Hare, 1988); for example, a meta‐ethnography exploring young people's experiences of engaging with mental health services generated novel insights regarding the silencing of suicide in clinical conversations and the absence of at‐risk groups' voices from the synthesised studies (Gilmour, Ring, & Maxwell, 2019). Although a meta‐aggregation of children and adolescents' perspectives on engaging with universal, depression and/or anxiety prevention interventions focused on insights relating to the most beneficial intervention components and barriers (Bastounis, Callaghan, Lykomitrou, Aubeeluck, & Michail, 2017), no experiences with PSSP interventions were synthesised. Notably, no qualitative synthesis of adolescents' experiences with PSSP exists.

Our epistemological stance guided the use of a meta‐ethnographic approach to address the research objective. The critical paradigm reflects our assumption that individuals exist within power‐laden societies and ultimately, power and inequity permeate into all aspects of collaboration and autonomy (Leavy, 2017; Scotland, 2012). There are unavoidable power imbalances between participants and researchers (Leavy, 2017; Scotland, 2012) which are amplified in research involving young people (Facca, Gladstone, & Teachman, 2020); for the most part, young people's experiences are presented through adult lenses (Montreuil, Bogossian, Laberge‐Perrault, & Racine, 2021). Moreover, power imbalances are palpable in schools given the unavoidable power hierarchies; teachers assume instructive and authoritative roles whereas adolescents are more passively positioned (Kohfeldt, Chhun, Grace, & Langhout, 2011). As such, consideration of power is of utmost importance in school‐based research.

Evidently, youth voice is a complex construction situated in multiple interpretations, which are influenced by power (Facca et al., 2020), and thus, exploring adolescents' experiences and perspectives while recognising data as context‐bound and power‐laden is needed (Braun & Clarke, 2019; Campbell et al., 2011). Meta‐ethnography is an interpretative, qualitative synthesis methodology which can generate novel insights and identify absences or obscurities in knowledge (Campbell et al., 2011; Mays et al., 2005), and is a particularly suitable qualitative synthesis methodology for our review.

### Objective

The objective of the meta‐ethnography review is to explore and integrate the perspectives and experiences of adolescents engaging with PSSP interventions, as participants/end‐users, intervention advisors, facilitators and co‐designers and co‐researchers.

## Methods

A published protocol for this review is available outlining the methodology presently employed (Walsh, Herring, & McMahon, 2023). This review was preregistered with PROSPERO (ID: CRD42022319424). Minor protocol amendments are reported in Table S1. The meta‐ethnographic approach followed Noblit and Hare's (1988) seven‐stage process for conducting meta‐ethnography and meta‐ethnographic procedures (Campbell et al., 2011; France et al., 2019; Lee, Hart, Watson, & Rapley, 2015). This review adhered to the PRISMA statement guidelines for systematic reviews and the eMERGe reporting guidance for meta‐ethnography (France et al., 2019; Page et al., 2021).

### Seven stage process for conducting meta‐ethnography

#### Stage (1): Getting started

The Introduction section outlines the identified topic of interest for the review and the rationale for the use of meta‐ethnography as an evidence synthesis method.

#### Stage (2): Describing what is relevant to the initial interest

The search strategy identified studies to be retained (Noblit & Hare, 1988). The search strategy can be found in Walsh et al. (2023) and is summarised herein. Eligible studies included adolescents aged 11–19 years engaging with PSSP interventions (as participants/end‐users, intervention advisors, facilitators co‐designers and co‐researchers) situated in post‐primary schools, targeting suicide‐related outcomes as both primary and secondary intervention outcomes. Studies that include a method of qualitative data collection or analysis to report on adolescents' perspectives of engaging with interventions were eligible for inclusion, including qualitative and mixed‐method studies. Studies including interventions located in third‐level education or university settings were excluded. In April 2022 and January 2023, PsycINFO, MEDLINE, Web of Science, CINAHL, ERIC, Scopus, ProQuest, British Library EthOS and DART‐Europe E‐theses Portal were searched to identify studies and grey literature published from database inception. Google Scholar and identified studies' reference lists were also searched. The first author assessed study eligibility based on title and abstract. Eligible full texts were assessed in duplicate by the first and second authors using Rayyan (Ouzzani, Hammady, Fedorowicz, & Elmagarmid, 2016) and disagreements were discussed until consensus was reached. Cohen's Kappa coefficient (Cohen, 1960) indicated adequate agreement on original full‐text screening yes/no decisions (*κ* = .69, *p* < .001).

#### Stage (3): Reading included studies

The first author led the coding generation and data analysis. The first and supervising author met on an ongoing basis throughout the coding process and analysis to discuss and refine the codes, concepts and overarching constructs in the context of important epistemological study concepts, including power and youth voice. Power and youth voice were discussed in the context of their positioning within the sample of studies, research and school contexts and in wider society. Ultimately, the supervising author provided guidance and critique throughout the data generation and analysis process to collaboratively develop meta‐ethnographic interpretations. Active reading and re‐reading of study data involved annotation and coding of constructs. Data relating to adolescents' perspectives on and experiences with PSSP interventions, including first‐order constructs (i.e. participants' quotations) and second‐order constructs (i.e. study authors' insights) from study result and discussion sections, were extracted. Other information from studies was also extracted, including study, participant and intervention characteristics, and is presented in Table S2. Three studies were extracted in duplicate by the first and second author using a detailed data extraction protocol. There was an 88.46% proportion of agreement (Gisev, Bell, & Chen, 2013) between the data simultaneously extracted from the three studies, and therefore, the data from the remaining studies were each extracted by either the first or second author. As outlined in Table S1, we included studies that partially lacked conceptual depth in the present meta‐ethnographic review.

#### Stage (4): Determining how the studies are related

First, first‐ and second‐order constructs within studies were line‐by‐line coded according to *how* adolescents were engaged in PSSP interventions using NVivo12. From herein we refer to these codes as concepts. Prominent concepts and within‐study relationships between concepts were identified. Between‐study comparison of concepts and within‐study relationships led to the development of initial conceptual categories representing common and recurring concepts sharing underlying central ideas and metaphors. These processes were iterative in nature and involved re‐reading and refining concepts and their relationships with one another within and across studies. Data were fully integrated to represent adolescents' experiences irrespective of how they were engaged in PSSP interventions, similar to procedures by Evans and Hurrell (2016). Studies were organised based on the presence or absence of the conceptual categories for analysis. Study characteristics including research design, intervention characteristics and participant characteristics were subject to cross‐study comparison. Study characteristics and findings were largely similar to one another, which informed the decision to conduct reciprocal translation (Noblit & Hare, 1988).

#### Stage (5): Translating the studies into one another & stage (6): synthesising translations

Stages (5) and (6) were interweaving, with resulting translations and synthesis iteratively engaged with. Reciprocal translation enabled the making sense of the data through the direct translation of similar concepts within the grouped studies into each other through a process of constant comparison, while retaining the characteristics of the studies. For example, the concept ‘want for the YAM intervention to include real life stories of young people's mental health experiences’ was translated into a similar concept ‘Suggestion for more personal mental health and help‐seeking experiences to improve the Silence is Deadly intervention’ to form a concept embodying these concepts titled ‘Need for PSSP interventions to include young people's mental health stories, ranging from personal experiences to seeking help’. This process resulted in third‐order (i.e. review authors) rounded constructs comprised of concepts embodying multiple concepts. Furthermore, findings were further abstracted, linked and interpreted to develop a broader interpretation of the constructs, which goes beyond the narratives reported in the individual studies, which are represented in a LOA synthesis (Campbell et al., 2011; Noblit & Hare, 1988; Toye et al., 2014).

#### Stage (7): Expressing the synthesis

This meta‐ethnography is best expressed as an academic publication in a journal that targets both researchers and practitioners in adolescent mental health fields.

### Study quality appraisal

Study quality appraisal was conducted using CASP checklists (CASP, 2013) undertaken in duplicate by the first and second authors and a research assistant. An additional question was added: ‘Are the interventions of interest clearly described?’ Disagreements in CASP checklists were discussed until consensus was reached.

## Results

### Characteristics of retained studies

**Figure 1:**
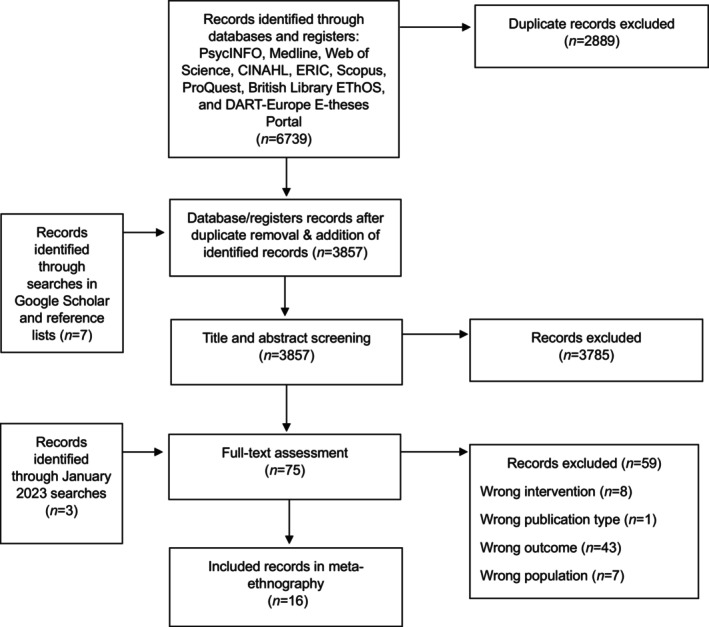
Meta‐ethnography PRISMA 2020 flow diagram

Sixteen studies published between 2010 and 2022 were retained (see Figure 1). Table S2 summarises the findings from the retained studies, which were mostly journal articles (three studies were dissertations). Table S3 presents the CASP assessments.

Overall, studies sampled approximately 3000 participants (not all studies reported exact sample sizes). Sample sizes for qualitative and mixed methods studies ranged from *n* = 5–1196. Sample sizes for qualitative studies ranged from 5 to 60 participants. For the 10 studies reporting age, participants were aged 12–19 years. Most studies included mixed‐gender samples, except for one study, which sampled only males and evaluated a male‐targeted intervention; four studies did not report gender. Studies were mostly conducted in North America (8/16 studies), Europe and Asia (each 3/16) and Australia (2/16). Most qualitative analyses of adolescents' experiences and perspectives were explored as part of studies evaluating interventions using mixed‐method approaches (11/16 studies). Most (10/16) studies collected data via focus groups, with surveys (5/11) and interviews (6/16) also being employed as data collection strategies (three studies employed several data collection approaches).

PSSP interventions contained the following levels of preventive strategies: (a) universal (12/16 studies), (b) selective (4/16), and (c) indicated (3/16). Signs of Suicide (SOS) was the most commonly cited intervention both evaluated in full or informing intervention development (3 studies). Most studies evaluated interventions that targeted STBs through risk and protective processes and health and well‐being outcomes, including knowledge, attitudes and norms impacting help‐seeking behaviour for STBs; resilience; body image; bullying; substance abuse; and cultural identity; and school‐oriented targets, including school climate and school engagement. Adolescents were mostly engaged as end‐users/recipients (11/16 studies). Three studies each engaged adolescents as peer helpers who helped to identify peer suicide risk and as co‐creators who co‐created prevention videos and interventions.

### Synthesis and analysis

Reciprocal translations and synthesis generated three overarching constructs.

#### The importance of trust in engagement with PSSP interventions

This construct conceptualises the importance of trust in participants' engagement with interventions. Trust was a concept that was both explicitly reported on and was inherently important for participants' engagement in several studies. Given the sensitivity of suicide and mental health topics, it is unsurprising that trust (or lack of) in individuals involved in interventions and school settings was integral for intervention engagement.

Trust in aspects of the school context, including school personnel, peers, and the wider school setting was inextricably linked to participants' perceptions of interventions. Trust in teachers was important in both teacher‐delivered and external facilitator‐delivered interventions. Where teachers delivered interventions (Klim‐Conforti et al., 2022; Shinde et al., 2017), participants described student–teacher relationships marked by both a lack of trust and hesitation in reaching out to teachers. Similarly, in external facilitator‐delivered intervention studies (Quinn, 2019; Wasserman et al., 2018), participants reported their preference for having external facilitators due to the perception that external facilitators are more respected, trained in the relevant topics, and allow for more youth‐led contributions.

It was important for participants to perceive facilitators as knowledgeable, skilled and open about mental health and suicide (Chartier et al., 2022; Johnson, Hart, Rossetto, Morgan, & Jorm, 2021; Labouliere, Tarquini, Totura, Kutash, & Karver, 2015). Moreover, trust and confidence in the ability of intervention facilitators were integral to how participants perceived PSSP interventions. One participant shared how having an open facilitator made it easier to engage with the intervention: ‘I think the way they approached everything and how they didn't tiptoe around issues made it easier to learn and understand.’ (Johnson et al., 2021). Conversely, a perceived lack of comfort in facilitating teachers (Klim‐Conforti et al., 2022) and participants themselves (Shinde et al., 2017) hindered participants' intervention engagement.

Furthermore, trust in peers engaging in interventions was integral for participants' engagement. Participants developed trust with peers from interacting during the interventions (Klim‐Conforti et al., 2022; Ohlmann, Kwee, & Lees, 2014; Robertson, 2008; Zachariah, de Wit, Bahirat, Bunders‐Aelen, & Regeer, 2018). Working in peer groups facilitated this building of trust and interaction, and beyond these studies, working in small groups was nonetheless recommended (Johnson et al., 2021; Labouliere et al., 2015). Positive experiences of engaging with interventions due to a sense of trust fostered by interacting in small groups are conveyed as follows:

The impact the LifeSavers training had on me was my trust in others. In the listening circles, I didn't know if I could just trust a strange group of people. As the activities went on, I was able to trust them. I actually trust my group more than my friends. (Robertson, 2008)

However, some participants felt that their schools were not prepared to host interventions due to the inclusion of topics which are typically ‘out of bounds’, such as suicide (Johnson et al., 2021; Klim‐Conforti et al., 2022). One participant conveyed how a lack of trust in schools to deliver interventions could impact on engagement: ‘School never talks about it (mental health), you can't bring it up one time and expect us to talk about it. I just do not trust them.’ (Klim‐Conforti et al., 2022).

#### Different to engaging in school: The importance of how adolescents engage with PSSP interventions

This construct makes sense of the impact of *how* participants are engaged with PSSP interventions on their intervention experiences. Participants did not want to be passive recipients of interventions and valued active engagement in interventions. Participants reported largely positive experiences with experiential and agentic engagement (Braun, Till, Pirkis, & Niederkrotenthaler, 2021; Chaniang, Fongkaew, Stone, Sethabouppha, & Lirtmunlikaporn, 2019; Klim‐Conforti et al., 2022; Ohlmann et al., 2014; Quinn, 2019; Shinde et al., 2017; Wasserman et al., 2018; Wexler et al., 2017). Participants and authors reported on the various benefits of discussions, role plays and debates, including facilitating learning and sustaining interest in interventions. Agentic engagement included leading the intervention focus, collaborating to develop and implement interventions, voicing questions and concerns and outreach activities such as creating suicide prevention interventions.

Participants had more positive experiences when interventions allowed for youth‐led discussions and open dialogue between participants and facilitators (Braun et al., 2021; Klim‐Conforti et al., 2022; Quinn, 2019). Participants juxtaposed their contentment with active engagement in the intervention with their typical didactic engagement in the classroom, as captured in the following excerpt:

Yeah, it was different to classes because it was a more open discussion…Yeah, we got to talk to each other more than when we're in class…it's not something you do, like, in a normal school class, so not too many people know much about it, or, how to think about this kind of stuff. So, that's why I think it's good. That's why it's useful. (Quinn, 2019)

Arguably, Braun et al. (2021) and Ohlmann et al. (2014) contained interventions which were among the most autonomously engaging, insofar that participants co‐created suicide prevention videos and presentations, respectively, for their schools and communities. Both studies reported transformative effects on participants, personal growth and enhanced confidence. One participant made sense of their experiences by comparing their normative experiences of being positioned as lacking in knowledge and autonomy by adults:

I really liked that adolescents were involved, because often adults believe, ‘adolescents don't even know what life is actually about’. In my opinion, we were treated as very mature and as adults, and in the same way we treated the subject matter in a very mature way as well. I really enjoyed that we were engaged on eye‐level. (Braun et al., 2021)

Similarly, participants in studies with greater didactic engagement nonetheless recommended more experiential engagement (Johnson et al., 2021; Klim‐Conforti et al., 2022; Labouliere et al., 2015; Mirick & McCauley, 2022), as captured in the following excerpt: ‘There was not enough activities that got us involved so many people may have zoned out. It's best to remember that we are high schoolers who need to get involved to stay awake.’ (Johnson et al., 2021).

#### Sharing of real‐life experiences important for connection and engagement

This construct captures the importance of sharing real‐life experiences and stories for connection and engagement with PSSP interventions. Personal experience sharing centres interventions on adolescents' own experiences, which further motivates meaningful engagement. Sharing of personal experiences related to mental health was reported as a particularly beneficial aspect of PSSP interventions in several studies (Braun et al., 2021; Calear, Morse, Batterham, Forbes, & Banfield, 2021; Johnson et al., 2021; Ohlmann et al., 2014; Quinn, 2019; Robertson, 2008; Wasserman et al., 2018; Zachariah et al., 2018). Personal experiences were shared via (a) listening to and talking about each other's experiences and (b) presenters/participants in person and in videos. Participants connected to the personal experiences shared, which piqued their interest in the intervention, led to personal development and built connection with others involved in the interventions. One participant shared their perception of the personal experience sharing as follows:

I think by visually watching the videos and examples of students our age experiencing these problems in life gave me a deeper understanding in Mental Health First Aid because I would listen to them and their feelings and understand as [a] friend how they would like to be treated and how I could help if it ever occurs. (Johnson et al., 2021)

Participants recommended even more opportunities for personal experience sharing for both themselves and individuals who had experienced mental health difficulties (Calear et al., 2021; Johnson et al., 2021; Quinn, 2019; Wasserman et al., 2018). Furthermore, participants who watched videos which depicted real life experiences (Hudson, 2016; Johnson et al., 2021) wanted more authentic and nuanced real life scenarios, with Johnson et al. (2021) stating that many participants recommended having a young person who had experienced a mental health crisis share their experiences. Furthermore, in Hudson (2016) participants identified the absence and need for real‐life suicidal behaviour experiences, with one participant noting the following:

I think it would have been like better and kids would have connected more if they heard about real stories of what actually happened to kids instead of just being like oh, it's not gonna happen but you know, just in case of like the 1% that it will happen. They should actually talk about real cases of when it happened. (Hudson, 2016)

Recommendations for greater personal experience sharing were also evident in studies, which did not contain these activities (Klim‐Conforti et al., 2022; Labouliere et al., 2015; Mirick & McCauley, 2022). One participant voiced wanting the opportunity to share their own perspectives, as follows:

After she (the teacher) was done talking, we, after a lot of people in our class started raising their hands, and then she said it was all done, you couldn't really tell someone else how you felt about it, because you felt, kind of, embarrassed to. And you didn't really want to raise your hand and say something. (Klim‐Conforti et al., 2022)

### Lines‐of‐argument synthesis

**Figure 2:**
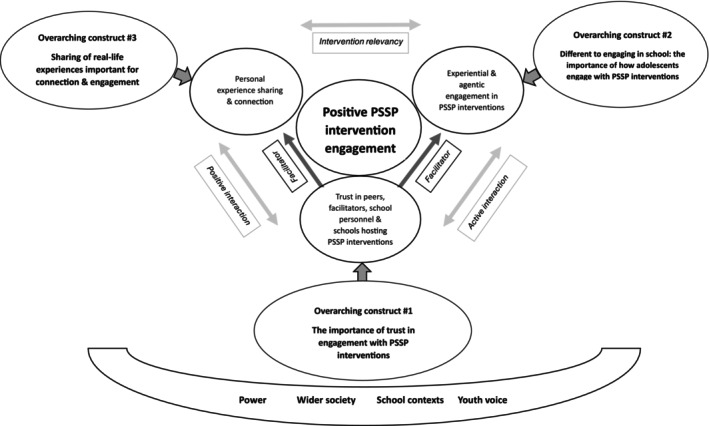
Lines‐of‐argument synthesis diagram

Figure 2 presents a diagrammatic narration of the LOA synthesis. LOA synthesis identifies cyclical relationships between the following: (a) trust in peers, facilitators, school personnel and schools hosting PSSP interventions, (b) experiential and agentic engagement in PSSP interventions, including leading the focus of interventions and collaborating to develop interventions and (c) personal experience sharing and connection. Trust was a central facilitator to the sharing of the self, building connection and experiential and agentic engagement, which collectively were key components of positive intervention engagement.

Engaging actively in PSSP interventions disrupted the typical positioning of participants as recipients of adult‐led didactic instruction. Personal experience sharing was identified as an intervention component of central importance, as it ensured both the relevancy of and interest in the activities and topics discussed and connected participants to each other and the interventions in which they were engaged. Moreover, trust was particularly important in interventions that required experiential and agentic engagement. The sensitive nature of topics and personal experience sharing in PSSP interventions can render participants vulnerable, which is amplified by (a) participants largely engaging in interventions in groups, and (b) participants having pre‐existing and sometimes negative perceptions of school contexts, in which PSSP interventions are ingrained. As such, trust in individuals involved in PSSP interventions and the wider school setting is integral for participants to engage in interactive and personal ways. In some studies, trust led to the sharing of the self and connection and vice versa, which underscored the positive experiences of adolescents with interventions. Trust, connection and overall positive intervention experiences were synergistically empowering and transformative, which is evident by the findings that participants (including at‐risk adolescents) in studies incorporating agentic engagement led outreach activities to help others beyond their trusted and connected groups.

## Discussion

A systematic review subject to a meta‐ethnographic approach employed reciprocal translations and LOA synthesis to explore and integrate adolescents' experiences of engaging with PSSP interventions as end‐users, co‐creators, and peer helpers. Our review addresses an important gap in understanding adolescents' experiences of engaging with PSSP interventions. The generated constructs reveal the importance of the following: (a) trust in individuals and schools involved in interventions, (b) *how* adolescents are engaged in interventions and (c) sharing of real‐life experiences for both connection and intervention engagement. LOA synthesis interlinks these constructs to provide a novel narration of adolescents' experiences with PSSP interventions. Adolescents' trust and connection in interventions were notably important for personal experience sharing and actively engaging in interventions, which included co‐design and outreach activities. Finally, the impacts of PSSP on adolescents are influenced by the ingrained nature of interventions in school settings, demonstrating the intersection between school contexts and adolescents' intervention experiences.

The present findings demonstrate the potential for (a) poor adolescent‐school personnel relationships and (b) a lack of trust in individuals and school settings to undermine adolescents' intervention engagement. Our findings reflect previous findings relating to adolescent help‐seeking for mental health, with key help‐seeking barriers including concerns about trust and confidentiality and perceptions of adult helpers as unhelpful and untrustworthy (Cigularov, Chen, Thurber, & Stallones, 2008; Gulliver, Griffiths, & Christensen, 2010). Furthermore, the review by Gulliver et al. (2010) identified links between stigma and concerns about both confidentiality and trust, insofar that peers and family could find out about young people's help seeking, which similarly could provide an explanation as to why a lack of trust in school personnel and schools could hinder adolescents' engagement in PSSP interventions. Taken together, these findings contradict the notion that students are more likely to prefer school personnel‐delivered school‐based mental health interventions given the daily contact and rapport between students and school personnel (Bastounis et al., 2017). Although it may be true in the context of strong and trusting student–school personnel relationships, it is not a given in all circumstances.

Our findings demonstrate adolescents' preference for PSSP activities, which are experiential, and resonate with findings in the wider school mental health intervention field that young people prefer hands‐on practical activities in comparison with didactic activities (Foulkes & Stapley, 2022). Montero‐Marin et al. (2022) conducted a secondary analysis to explore for whom the school‐based MYRIAD study worked/did not work (*N* = 8376). Despite good fidelity, reach and dosage of the intervention, participants' engagement was strikingly low, leading the authors to conclude that more engaging pedagogical approaches may have enhanced the accessibility, engagement, and effectiveness of the intervention. Moreover, our findings show that adolescents' engagement with PSSP interventions was influenced by peers in both positive and negative ways. This is unsurprising, given that adolescents are developmentally more susceptible to peer influence in comparison with their non‐adolescent counterparts (Ciranka & van den Bos, 2019; Prinstein & Dodge, 2008). Moreover, when engaging in classroom‐based mental health interventions, adolescents can feel vulnerable sharing private thoughts and feelings in front of peers (Foulkes & Stapley, 2022). In a study exploring the implementation of ‘Welcome To School’, a classroom‐based, teacher‐led psychosocial intervention for migrant adolescents, the classroom rules (i.e. respect and confidentiality) were key for facilitating a confidential space where sharing of personal experiences among adolescents could occur (Borsch, Verelst, Jervelund, Derluyn, & Skovdal, 2023), which further aligns with the LOA synthesis highlighting the importance of trust and connection for agentic and experiential intervention engagement.

The present finding showing the importance of adolescents' sense of trust in school settings to host PSSP interventions aligns with findings that (a) educational settings' culture was key for effective social, emotional and mental well‐being provision (Demkowicz et al., 2023), and (b) supportive school environments encouraged adolescent peer researchers' engagement with *Health Promoting Schools* (Kontak, Caldwell, Kay‐Arora, Hancock Friesen, & Kirk, 2022). Furthermore, Kontak et al. (2022) identified that key barriers to engagement included both: (a) adolescent and adult power imbalances and (b) adolescents' perceptions of not being taken seriously. The importance of supportive and trusted school environments in facilitating intervention engagement needs to be considered in the context of the aforementioned unavoidable power differentials in schools.

### Strengths and limitations

To our knowledge, this is the first review to explore and integrate qualitative findings representing adolescents' experiences with engaging in PSSP interventions.

Our review identifies several knowledge gaps in the PSSP field, which align with our previous PSSP intervention review findings (Walsh, Herring, & McMahon, 2022). First, our review contains largely white samples located in predominantly white countries. Although the aim of qualitative research is not to be representative, this knowledge gap is similar to school‐based mental health qualitative research more generally, which has mostly sampled white and middle‐class participants (Foulkes & Stapley, 2022). This is problematic given that suicide risk peaks in adolescence and young adulthood in ethno‐racially minoritised young people in comparison with their white counterparts (Alvarez, Polanco‐Roman, Breslow, & Molock, 2022). Second, there was a lack of consistency in the reporting of important contextual factors, including participant and school demographics. Of note, six of the identified studies did not report age, despite age being a critically important demographic variable in adolescence, school settings and PSSP interventions. Taken together, these identified gaps highlight the urgent need for (a) more research exploring perspectives on PSSP interventions among minority adolescent samples, and (b) enhanced reporting of contextual characteristics in PSSP intervention studies.

In terms of sampling, most of the retained mixed‐method studies sampled a subgroup of the participants for the qualitative component of the intervention evaluation, often utilising a self‐selecting recruitment method. Foulkes and Stapley (2022) note that the qualitative evaluation of school mental health interventions can include samples largely representing adolescents who are more enthusiastic about the intervention or discussing their participation. For instance, the majority of the participants who drop out of the earlier stages of PSSP intervention trials are not included in the end‐of‐intervention qualitative evaluation, which likely results in findings that are more favourably inclined towards interventions. Foulkes and Stapley (2022) recommend asking all participants to numerically rate how much they liked an intervention, which could provide researchers with information to guide their invitation for follow‐up inquiry to participants with a diverse range of opinions.

Most studies were of good quality, though there were systematic failings in considering the relationship between researcher and participants. Given the aforementioned importance of considering power imbalances in school‐based research, advocacy/participatory worldviews and critical perspectives, which recognise the importance of power and the positioning of researchers and adolescents in research and wider society (Creswell & Poth, 2016), should guide future PSSP research and wider school mental health research.

Refutational translation is an important meta‐ethnographic synthesis method, which can be often overlooked (France et al., 2019). We did not include refutational translations in the present review given that the identified studies in our review were largely similar rather than dissimilar and mostly contained notable and meaningful similarities. Team size and data volume are known factors that influence data analysis decision‐making (France et al., 2019). Pragmatically, we had to balance having a small team with the volume of data that needed to be processed, analysed and synthesised. Ultimately, the laborious data analysis involved in meta‐ethnography requires extensive and iterative engagement as part of a collaborative analytic process. Taken together, these factors informed our decision‐making to focus the analysis on reciprocal translation and LOA. Our analytic decision aligns with recommendations by Campbell and colleagues and Noblit and Hare for conducting meta‐ethnographic reviews.

The literature searches for the meta‐ethnographic review were conducted in January 2023 and searches of databases were repeated in March 2025. A total of 3336 records were identified from this search and were subject to screening based on title and abstract. Four records were retained for full text analysis, and two of the records did not meet the inclusion criteria; Panagioti et al. (2023) and Meena et al. (2024) did not report on qualitative methods or findings exclusive to adolescents aged 11–19 years. The records that did meet the inclusion criteria were co‐authored by Morris (2024) and Gaete et al. (2025). The sample in our review is purposive, which aligns with our aims to generate interpretative explanations rather than predictive explanations based on an exhaustive sample of studies (Thomas & Harden, 2008). For these reasons, the comprehensiveness of literature searches is generally not a desirable goal of qualitative synthesis reviews, nor is it attainable, given the large volumes of data that form the basis for the analysis (Lorenc et al., 2012). In our review, the study characteristics and findings were largely similar across studies, and as such, it is likely that there would have been diminishing returns in terms of extra insight or validity from the inclusion of more eligible studies (Lorenc et al., 2012), nor would it have been pragmatic. However, we include a summary paragraph below describing the two studies that met the inclusion criteria.

Gaete et al. (2025) report on an evaluation of the Reframe‐IT + intervention, which is a blended internet‐ and school‐based CBT program aiming to reduce suicidal ideation among adolescents. Adolescent participants from 6 public schools in Chile (*n* = 52) were randomised to receive Reframe‐IT + (*n* = 33) and TAU control (*n* = 19). Qualitative data were collected using a mixed‐method survey to assess intervention acceptability, which was then categorised into content, pedagogy and logistics categories. Qualitative findings show that Reframe‐IT + helped adolescents to identify, express and manage their emotions and enhanced problem‐solving and help‐seeking. Adolescents liked the intervention activities and resources, including videos, the message board, brochures and the mood diary, as well as the facilitators with whom they built relationships. However, adolescents voiced the need for intervention components, including the platform and flyers, to be more creative and for sessions to be more personalised and longer in length. More opportunities to communicate thoughts and receive targeted help were also noted. The second record authored by Morris (2024) is a doctoral dissertation that contains a qualitative study examining the experiences of high school students in the US who engaged with the Hope Squad program, a peer‐based suicide prevention programme. Seven students participated in a focus group or interview, and data were analysed using thematic analysis. The findings demonstrate that the Hope Squad programme was received positively by participants, and engaging with peer tutors was a comfortable, relatable and empathetic experience aiding learning and growth. However, some participants noted perceiving some of the peer tutors as nervous in their program delivery. Moreover, participants expressed the value they gained from personal experience sharing by their peer tutors, which aided participants in understanding how to apply the programme learnings in their own lives. Participants also noted that they learned valuable lessons beyond the program curriculum, including leadership skills. Taken together, these findings largely align with the reciprocal translations and LOA synthesis findings.

### Implications

Trust, connection, personal experience sharing and agentic engagement were key to adolescents' empowering and transformative PSSP intervention engagement, and therefore should be considered important concepts for youth‐centred PSSP intervention design and implementation priorities. Moreover, in our review, most studies employed universal interventions, which involve participants often engaging with the same content in the classroom setting. However, some participants may not find the intervention particularly relevant or helpful to them, or worse, may be harmed by these interventions (Foulkes & Stringaris, 2023). Considering the challenge of balancing the usefulness of universal interventions' widespread reach with the need for these efforts to be beneficial to all, our finding that personal experience sharing enhanced intervention relevancy is a key finding for both the PSSP and wider school mental health fields. We recommend greater consideration of universal PSSP interventions, which centre interventions on adolescents' experiences and viewpoints in ways that recognise that adolescents will have varying interests and capacities to share and receive personal experiences.

It is integral that due consideration is given to relationships among adolescents participating in PSSP interventions to ensure that interventions can reach their full potential. Moreover, facilitators' and teachers' roles in PSSP interventions and relationships with adolescents are modifiable factors which can both be harnessed to maximise adolescents' intervention engagement. We recommend the exploration of these concepts from the perspectives of adolescents as an important direction for PSSP research. In practice, adolescents engaging in PSSP interventions should have opportunities to (a) engage in relationship‐building activities, (b) engage with intervention components in smaller groups and (c) opt out of activities.

Our findings highlight a clear gap in PSSP intervention research, which employs participatory designs, with only 3 out of 16 of the studies engaging adolescents as co‐creators in aspects of the interventions. Participatory research can be particularly complex and resource‐intensive in school settings for several reasons: (a) there can be a dichotomy between adolescents' positioning in the school settings versus the active involvement of adolescents in participatory research (Kohfeldt et al., 2011), (b) there are notable complexities related to marrying interventions with classroom and school contexts (Borsch et al., 2023; Kohfeldt et al., 2011) and (c) there are various PSSP intervention stakeholders to consider in comparison to non‐school based research. As such, there is a need for greater evidence of the effectiveness of school‐based suicide prevention interventions that are co‐created with young people and the implementation potential of these interventions in school environments.

## Conclusion

Our review makes an important contribution to the field of PSSP by presenting novel insights into the importance of exploring and integrating the perspectives and experiences of adolescents engaging with PSSP interventions. Going forward, PSSP research needs to focus on PSSP intervention effectiveness and implementation matters pertaining to both the experiences of adolescents who are at the centre of PSSP interventions and the schooling contexts in which PSSP interventions are situated. Our findings transcend the field of PSSP and are relevant to wider research fields and adolescent mental health practice, which value youth voice and youth engagement.

## Funding

There are no funders to report.

## Conflict of interest

The authors have declared that they have no competing or potential conflicts of interest.

## Ethical considerations

No ethical approval was required for this systematic review.

## Trial registration

This review was preregistered with PROSPERO (ID: CRD42022319424). https://www.crd.york.ac.uk/PROSPERO/view/CRD42022319424. The date of registration in PROSPERO was 19 April 2022.

## Supporting information


**Table S1.** Amendments to protocol for the meta‐ethnography.

**Table S2.** Summary of retained study characteristics and findings.
**Table S3.** CASP checklist scores for retained meta‐ethnography studies.

## Data Availability

Data sharing is not applicable to this article as no new data were created or analysed in this study.
