# Supplementary material for: Review: Adolescents' perspectives on and experiences with post‐primary school‐based suicide prevention as end‐users, co‐creators and peer helpers – a systematic review meta‐ethnography
Source: Child Adolesc Ment Health. 2025 Nov 6;31(3):217–28. doi: 10.1111/camh.70043 (PMC13474300; doi:10.1111/camh.70043)
Supplement: Supplementary file 1 [file CAMH-31-217-s001.docx]

**Supporting Information**

**A meta-ethnography of adolescents’ perspectives on and experiences with post-primary school-based suicide prevention as end-users, co-creators, and peer helpers**

Eibhlin H. Walsh,^1,2,^ Laura Hemming,^3^ Matthew P. Herring,^4,5,^ Jennifer McMahon^1,2^

^1^School, Child & Youth (SCY) Mental Health and Wellbeing Research Lab, Health Research Institute, University of Limerick, Limerick, Ireland

^2^Department of Psychology, University of Limerick, Limerick, Ireland.

^3^Violet Vines Marshman Centre, La Trobe Rural Health School, Melbourne, Australia

^4^Physical Activity *for* Health Cluster, Health Research Institute, University of Limerick, Limerick, Ireland	^5^Department of Physical Education and Sports Sciences, University of Limerick, Limerick, Ireland

|  | **Supplementary Table S2** | | | | | | | |
| --- | --- | --- | --- | --- | --- | --- | --- | --- |
|  | *Summary of Retained Study Characteristics and Findings* | | | | | | | |
|  | **Authors** | **Sample** | **Intervention** | **Study Aim & Methods** | **Key study categories/themes** | **Key meta-ethnographic concepts** |  |  |
|  | **Braun et al. (2021)** | Vienna, Austria.  *N*=17^a^ aged 15-19 years (*M*=16.4 years) 72.2% female. Co-creators of suicide prevention videos. | Creation of suicide prevention videos as part of an elective Suicide Prevention module. Personal experiences with crisis situations & coping strategies were also discussed & reflected on. Videos were presented to school personnel, parents & students at an evening event. Some videos would be later tested in a randomized controlled trial & used by suicide prevention organizations targeting youth). | Aim: to explore adolescents’ experiences of producing suicide prevention videos targeting other adolescents in a school setting using a participatory qualitative research approach. All participants interested in engaging with module were accepted for participation. Documentary method analysed data collected using focus groups. | Findings were clustered into five categories based on the four topics predefined for the interview guide (one additional category was identified during analysis): (1) Experiences During the Production Process Benefits; (2) Challenges Learning Effects, (3) Content of the Videos and Underlying Ideas, (4) Opinions on the Possible Effects of the Videos & (5) implications for Suicide Prevention | Important for co-creators to create videos that were relevant to adolescents  Appreciation for active involvement in the project  Pride in the created videos  Enhanced knowledge of STBs and associated stressors  Personal growth linked to engagement in the project  Appreciation for combining mental health and arts-based methods | |  |
|  | **Calear et al. (2021)** | Australian Capital Territory. *N*=222^a^ aged 16-18 years, grades 11 & 12. All male. | The Silence is Deadly is an upstream, public health program which addresses knowledge, attitudes & norms purported to impact on help-seeking behaviour for emotional problems & suicide. The psychoeducational program is delivered according to Menslink’s usual procedures & incorporates a presentation, website, videos, handouts & incorporates male role modelling & social norming. Key statistics about mental health, suicide, help-seeking & links between masculine norms & help-seeking are presented & discussed. It is delivered in partnership with trained local celebrity athletes, who appear as guest presenters & are included in a video speaking about their experiences. | Aim of qualitative component: to assess satisfaction with the program & explore the impact of the intervention on school culture. Framework analysis approach of data collected via survey. | No overarching categories reported. Mostly positive feedback on the program, **s**ome students did not enjoy the program. Participants found the program engaging, informative & easy to understand. Participants with similar mental health experiences to those described by the presenters connected to the presenters, conversely participants who did not share these experiences/ interest in sports did not relate as well to the presenters. Participants wanted more & a variety of personal stories | Appreciation for personal stories related to mental health  Lack of similar experiences between participants and presenters hindering relatability of presenters | |  |
|  | **Chaniang et al. (2019)** | Chiang Mai province, Thailand. *N*=30 & 12 Core Working Group who were peer leaders who collaborated to develop & implement the program. Grades 7-12^b^. ^c^ | This universal program aimed to prevent suicide & was built on previous programs (i.e., Lifeline Program & SOS) & the contents, strategies & activities were based on needs assessment & action research framework. The overarching study comprised of 3 phases: (1) situational analysis of school suicide prevention, interviews with parents/ teachers & focus group discussions with students, (2) collaborating with an adolescent Core Working Group to design & implement the program, including conducting a campaign of suicide prevention in school & revising the plan until the program was appropriate for the school context. Additionally, a family nurse conducted training sessions with parents on topics including adolescent development, parenting styles & parent-teen communication & a mental health nurse conducted a seminar on counselling with teachers. Adolescent Peer Leaders’ module (i.e., adolescent peer leaders’ enhancement & awareness for suicide prevention & fostering mutual support by adolescent peer leaders). An “edutainment” approach was used (i.e., games, role playing, and video clips). Parents’ & Teachers module (i.e., suicide prevention awareness & education & fostering mutual support by family) (3) the program was evaluated. | Aim of qualitative component: to evaluate a pilot suicide prevention program & evaluate the feasibility & appropriateness. Action research design. Thematic analysis of data collected using focus groups. | Analysis resulted in two themes & two sub-themes: (1) Improving suicide knowledge and attitudes & (2): It’s such a great campaign (sub-theme 1: It’s feasible & sub-theme 2: It’s appropriate) | Enhanced confidence and leadership skills from engaging in the peer leader group  Appropriateness of the intervention for participants and schools  Peer leaders’ improved knowledge and attitude towards STBs | |  |
|  | **Chartier et al. (2022)** | Manitoba, Canada.  *N*=30.  6 boys, 24 girls. | PAX Dream Makers is a youth-led addition to PAX-GBG (an evidence-based programme suicide prevention program), which engages middle & high school students to create peace, productivity, health & happiness in their schools & communities. It applies the strategies of PAX-GBG to support & empower Indigenous adolescents. This approach included special training through youth gatherings & ongoing support for youth & adult mentors to develop leaderships skills & enhance their understanding of the PAX-GBG programme. Dream Makers Gathering comprised of youth & adult mentors learning about PAX-GBG, youth creating a vision for their schools & communities, youth sharing their school & community visions & accomplishments while developing confidence & trust in each other & in their abilities & mentoring new Dream Makers with guidance from the PAX-GBG trainers. | Aim: To examine youth perspectives on the PAX Dream Makers approach. Case study design.  A line-by-line thematic analysis of data collected via focus groups & one on one interviews. | Findings were organised into the following themes, many of which were determined in advance based on the interview: (1) Positive influence of Dream Makers approach on youth, (2) Dream makers gatherings: strengths and suggestions, (3) Qualities of good mentors, (4) Challenges and suggestions to the PAX dream makers approach and (4) Empowerment through culture (sub-themes: Community context & Strengths and challenges to PAX-GBG implementation). | PSSP positively impacting on adolescents  Importance of having good adult mentors for intervention success | |  |
|  | **Hudson (2016)** | Boston, US. *N*=10 (a sample of participants who do not report changes in knowledge, attitude and/or behaviour). Grade 7.  55% female^b^ | The middle school version of the SOS program aims to reduce suicidal behaviour by increasing adolescents’ understanding of & ability to promote more adaptive attitudes toward depression & suicidal behaviour. Participants are taught ACT in the suicide awareness curriculum. In the didactic component, participants are taught to recognize signs of suicidality & depression in themselves and others. Participants are taught the appropriate action steps for responding to those signs. Curricular materials consist of a discussion guide and a video, the latter which includes dramatizations that portray the signs of suicidality & depression and the appropriate ways to react to someone who is exhibiting these signs. The video also includes testimonials from people whose lives have been affected by suicide. The SOS program also includes screening students for depression & suicide risk & subsequent referral for professional mental health support. | Aim of qualitative component: To investigate the shortcomings of the SOS program for participants who do not report changes in knowledge, attitude and/or behaviour?  Mixed methods.  Consensual Qualitative Research analysed data collected from one-to-one interviews. | The findings were organised into the following categories: (1) Helpful/positive, (2) Redundant, (3) Coping, (4) Real events, (5) Subtlety and (6) Other constructive feedback | Lack of intervention content which guides individuals on how to help themselves  Intervention contained information already known to the participants  Desire for content relevant and real to life  Perception that adolescents may not take aspects of the intervention seriously | |  |
|  | **Labouliere et al. (2015)** | Southwest, United States.  (*N*=58). 47% took part in the gatekeeper training).  9^th^-12^th^ grade^b^.  46.8% male. | The Jason Foundation “A Promise for Tomorrow” gatekeeper prevention program promotes awareness of youth suicide, provides participants with the knowledge & resources to interact with at-risk youth & develops positive attitudes toward referring at-risk peers. The training sessions were facilitated by teachers/counsellors during health classes & were overseen by school mental health personnel. | Aim of qualitative component: To enrich interpretation of quantitative results. Participants were randomly selected from the sample of the large evaluation study. Mixed methods approach.  Data from focus groups were  transcribed & responses were coded for themes. | No overarching themes reported. 64.5% of trained participants reported that recognition of suicidality in their peers remained difficult. Training had positive effects on participants’ confidence regarding talking with a suicidal peer. 45% of trained participants felt the program was informative, 53% reported that aspects of the program could be improved. Common recommendations for program improvement included having more interested & knowledgeable trainers in suicide prevention, enhancing program information & materials clarity, concreteness, and depth, increasing the frequency & degree of interaction (e.g., role plays, small group discussion) & updating the material with more ethnically diverse actors & current settings & situations. Generally, youth participants preferred program components that more closely approximate real-life settings in which they might be called upon to apply suicide prevention knowledge | Recommendations for intervention materials containing more relevant settings and situations  Recommendation for more experiential activities  Recommendation for trainers who are more interested and knowledgeable about suicide prevention  Perception that the intervention did not provide knowledge on how to support friend experiencing STBs  Intervention would benefit from smaller group discussions | |  |
|  | **Johnson et al. (2021)** | Melbourne, Australia. N=979. *M*=15.82 years. Year 10.  Female=43.94%. | TeenMHFA is an universal program which trains participants to support their peers who are experiencing a mental health problem or crisis (i.e., suicidal behaviours, bullying). The program was originally developed from findings derived from a large Delphi consensus study involving youth mental health experts & young people with lived experience during their senior school years investigating the provision of mental health first aid. The program meets the need for adolescents to be empowered to support their peers Content is delivered in three 75-min training sessions by an accredited & trained MHFA instructor. The classroom-based program is supported by audio–visual presentations, a student workbook, group activities & role plays. Facilitation of the program is manualized & all program materials have been refined with input from students, adolescents with lived experience of mental illness, educational experts & teachers. | Aim of qualitative component: to augment existing quantitative findings  by analysing qualitative feedback on the TeenMHFA  program provided by students at host schools, and to describe the experiences & opinions of participants through qualitative analysis of open-ended student responses to online survey. Mixed methods. Generalized content analysis, then a more detailed thematic analysis analysed survey data. | See Table II in Johnson et al (2021) for content analysis categories.  Findings were organised into the following themes relating to the strengths of the program: (1) connection with personal stories through visual media, (2) relevance of the practical nature of the course and (3) helping to promote mental health  understanding and awareness. Themes related to weaknesses include: (1) student engagement and (2) student participation and ‘buy-in’, and two themes for improvements: (1) more interaction & hands-on activities & (2) include student discussion and viewpoints. | Facilitators’ openness regarding mental health and suicide which is normally considered out of bounds in school  Hearing others lived experiences leading to connection  Recommendations for more adolescent lived experience sharing  Discussion of mental health topics during the intervention | |  |
|  | **Klim-Conforti et al. (2022)** | Toronto, Canada. *N*=60 (end-users). 12-14 years.  Grade 7 & 8. ^c^ | The 3-month universal, teacher-led program CBTSI aims to increase emotional regulation, resilience, and distress tolerance. While reading “Harry Potter and the  Prisoner of Azkaban,” students engage in discussion  about key learning objectives (i.e., identifying risk and protective factors, basic cognitive restructuring techniques, behavioural interventions to improve mood, and promoting help-seeking behaviour should these skills fail to improve distress tolerance. Students learn to recognize how depression and anxiety manifest in the characters in the novel & additional exercises augment student learning through a discussion of the symptoms, behaviours, & thoughts to recognize depression and anxiety in themselves and others.) Private workbooks, lessons (& a corresponding manual) Suicide will not be mentioned in the intervention, apart from a brief note that suicidal ideation can be a symptom of depression. Differentiated instruction modifies the learning experience to address the needs of participants who learn differently (i.e., adjusting content, exercises and practices and assessments. | Aim of qualitative component: to understand barriers & facilitators to engagement with the CBTSI. This mixed methods study draws on programme evaluation with a pragmatic approach. Analysis was informed using principles of pragmatism. Thematic analysis of focus group data. | Five themes & fifteen constructs were identified: (1) Confidence and self‑compassion, (2) Awareness, (3) Relationship to self and system, (4) Sense of community, and (5) Teaching and learning. | Trust resulting from developing connection during group activities  Experiential engagement leading to positive experiences engaging with the intervention  Importance of perceiving teachers as at ease with presenting the intervention for participant engagement  Teacher-student relationships and mental health focus in schools influencing intervention engagement  Desire for greater discussion of real life and relevant experiences | |  |
|  | **Mirick and McCauley (2022)** | USA. *N*=1,196.  6^th^–8^th^ (middle), 9^th^–12^th^ (high).  Female=654 (54.7). | Virtual SOS is a universal synchronous online program created in consultation with national suicidology & mental health experts. A school staff member delivered SOS to participants during classtime and participants logged onto video conferencing technology & attended the class from home. Virtual SOS differed in several ways from the in-person mode, including: (1) there was no screening component, (2) a web-based student response slip was developed, which allowed participants to submit their slip immediately following class, so that school staff had real time access to students’ responses and could provide the necessary support, screening & referrals. | Aim of qualitative component: to investigate: (1) if virtual SOS acceptable to middle & high school students? (2) How do middle & high school students perceive virtual SOS compared to in-person SOS? And (3) Are there differences in acceptability of virtual SOS between at-risk students (those who used SOS to ask for help for themselves) and students who are not at-risk? Mixed methods. Survey. Thematic analysis. | Themes are presented in tables 2, 3 & 4 under the following three headings: (1) Comparison of Virtual to In-Person, (2) Recommendation to Continue Virtual SOS, & (3) Ways to Improve Virtual SOS” | Mixed perceptions on in-person or virtual modes of delivery  Perceived discomfort in class-level discussions of suicide and self-harm topics  Recommendation to include more broad discussions and self-reflection  Recommendation to include more realistic and relevant examples | |  |
|  | **Ohlmann et al. (2014)** | British Columbia, USA. *N*=5, 15-18 years^c^. | The *Alive* group is an indicated youth-led suicide prevention education group for at-risk youth, with participants choosing the focus of suicide prevention. The *Alive* group involved the participants supporting one another and gaining skills and knowledge relating to mental health issues. Ultimately, the aim of the group was for participants to become literate in mental health issues, recognizing the signs of emotional distress & developing the skills needed to reach out to peers. The participants elected to create a presentation based on the “Ask, Assess, Act” model for other students, to teach other young people how to recognize signs of suicide risk in themselves & in peers. | Aim: to explore the insider accounts/experiences of the participants & to explore how resiliency was fostered through adolescents’ experience of a suicide prevention & education program. Listening Guide analysis of data collected from Focus groups & one-to-one interviews. | Analysis was organised into two overarching themes which contained sub-themes: (1) Voices of Vulnerability (Voice of not knowing, Voice of disconnection, Voice of silence & Voice of literal silence, and (2) Voices of Resilience (Voice of knowing, Voice of literal knowing, Voice of connection, Voice of altruism, Activist voice & Voice of protection). | Developing trust with others involved in the intervention facilitated sharing of experiences  Developing a sense of connection and belonging during the intervention  Pride and enjoyment in sharing insights with schools  Engaging in the intervention leading to enhance confidence and agency among participants  Personal story sharing leading to connection and empowerment with the group | |  |
|  | **Quinn (2019)** | Ireland, Dublin.  *N*=13, 10 females, 3 males.  Median age was 13 years^b^ 2^nd^ year. | The Resilience Academy program has a resilience-focused approach informed by CBT & DBT & is aimed at equipping participants with practical information & techniques to facilitate emotional & cognitive resilience. The program’s development was informed by evidence synthesis findings, industry experts and interviews with students and school staff in similar contexts to the one in which the intervention was evaluated. The upstream & universal program is facilitated by mental health professionals. Students initially learn about resilience, mental health & coping techniques. Participants choose from four modules (i.e., school stress & mental health) covered through discussion & activities. Handouts, story vignettes, slides, posters & videos are utilised by facilitators. | Aim of the qualitative component: to explore participants’ personal experience of the program & analyse the meaning of  change, the processes responsible for change & any recommendations for future programme development & implementation. Mixed methods design (randomly selected). Focus groups. Thematic analysis. | The findings are presented in the following themes & sub-themes: (1) A Positive School Experience (subthemes – Different to class, Opportunity to reflect and discuss), (2) Peer Connection (subthemes – Collaborative learning, Openness versus Anonymity), (3) Learning How to Cope (subthemes – Adaptive coping, Skills attainment) & (4) Student Recommendations (The Student Voice) (subthemes – Programme planning & Programme content) | Experiential engagement in the intervention different to typical engagement in the classroom  Peer interaction and connection facilitating intervention engagement  Positive experiences with sharing of and listening to stories and real-world experiences  Recommendations for more opportunities to reflect on and discuss mental health topics | |  |
|  | **Robertson (2008)** | Southern Illinois. *N*=82. Aged 14-18 years (*M*=15.33). Grade 9, 10, 11 & 12. 15 Male, 85 Female ^b^ | LifeSavers peer-helper training program is a peer-support, suicide & crisis-prevention training program, which involves training participants to be LifeSavers during a weekend retreat & continued training & support from school advisors. Participants are recruited to be LifeSavers through school presentations, staff/faculty recommendations & an essay detailing their interest & skills. Participants are selected if they show evidence of being trustworthy, open-minded, caring, respectful, responsible, & exhibits natural helper & leadership qualities. | Aim of the qualitative component: to explore the training’s impact, self-rated stress & suicide exposure. Mixed methods approach (participants who completed the surveys’ qualitative items were included). Survey. Content analysis. | Data was coded into the following categories: (1) Life-changing experience, (2) Emotional well-being, (3) Self-confidence, (4) Self-awareness, (5) Openness/Trust, (6) Helping/Listening skills, (7) Other-awareness/ Perspective-taking, (8) Home/Parent relationships, (9) Peer relationships, (10) General/Other relationships & (11) Education/ Suicide knowledge | Intervention engagement enhancing trust in participants and others more generally  Engaging with PSSP as a peer helper leading to improved helping and listening skills  Personal experience sharing leading to personal development and connection | |  |
|  | **Shinde et al. (2017)** | Bihar, North India. *N*=42, 13-18 years.  Grades 9 & 10. ^c^ | SEHER is an universal, whole-school health promotion intervention which targets school climate to promote adolescent health & wellbeing. SEHER activities include awareness generation activities, wall-magazine, competitions, speak-out box, school health promotion committee and health policies), peer groups and workshops. Individual-level supports include counselling & referral. SEHER aims to reduce substance use, sexual risk behaviours, bullying & violence, depressive symptoms. | Aim of the qualitative component: to gather youth perspectives about the intervention components & its delivery & to identify the gaps & improvements needed.  Mixed methods (schools were purposely selected based on high/low coverage of intervention activities). Focus groups. Thematic analysis. | The following analytical themes & subthemes were employed to the data (relevant to qualitative data related to young people are presented here): (1) Acceptability & (2) Feasibility (Facilitators to intervention delivery, barriers to individual-level activities). | Enjoyment of and recommendations for experiential activities  Discomfort in confiding in teachers regarding sensitive issues  Approachability of intervention facilitators important for engagement | |  |
|  | **Wasserman et al. (2018)** | Estonia, Italy, Romania, & Spain.  *N*=32, 15-17 years.  16 male & 16 female | The YAM programme is a universal intervention which promotes increased knowledge about mental health. YAM comprises of role-play sessions with interactive workshops & educational posters/booklets. It covers various mental health topics (i.e., depression & suicide). Youth input informs the intervention content & reflection is encouraged. | Aim: to have participants to speak about the YAM & mental health & understand their experiences with and thoughts on the YAM. Qualitative study (as part of a wider mixed methods study). One-to-one interviews were conducted (participants were randomly selected from the largest participating YAM schools in each country). A focus group & informal discussions with adolescents helped improve the format of the interview, including question development and language used. Grounded theory. | The following themes & sub-themes were identified: The YAM experience: (engaged: Recognizing emotions) Initially hesitant (Life experiences), eager to please (Positive feedback), Cautious (Not in front of others) disengaged (Indifferent perhaps). | Positive experiences engaging in experiential activities during the intervention  Positive experiences of listening to and sharing personal experiences and stories  Experiential activities facilitating personal story sharing  Recommendations for more lived experience sharing | |  |
|  | **Wexler et al. (2017)** | Alaska, United States. *N*=28 youth leaders & at least 10 youth captains. Youth leaders who participated in focus groups were mostly female. Cohort 1 = 6 (50%) female  Cohort 2 = 12 (75%) female. | Youth Leaders Program is a health intervention which targets youth suicide by reducing related risk factors (i.e., substance abuse & bullying) & increasing protective factors (i.e., school engagement and cultural identity). A 20-year partnership between communities and a university researcher relied on relationships and local guidance to steer the program evaluation. Youth leaders organize & host a variety of informational & recreational activities for students & their families, as well as assisting students. Youth leader captains are student leaders selected by peers & advisors from the participating youth leaders, who have at least one prior year’s experience in the program & have shown interest in & aptitude for leadership – they assist youth leaders. Prior to data collection, several youth leaders and other stakeholders involved discussed expected outcomes & the evaluation approach. | Aim of the qualitative component: to better understand how the program was being implemented across the district. Mixed methods approach. Grounded theory coding of data collected via focus groups. | The following categories related to young people were presented: (1) Youth leaders’ perceptions from focus groups, (2) Linkages Made Between Youth Leaders Program & Suicide Prevention & (3) Challenges Documented Through Focus Groups & Interviews with youth leaders & youth leaders advisors. | Helping others a beneficial outcome of the intervention  Positive experiences hosting activities as part of the intervention  Enhanced maturity from engaging with the intervention | |  |
|  | **Zachariah et al. (2018)** | India – Pune, Maharashtra. *N*=76. 12-15 years.  56 boys & 20 girls | The SALT approach & dream building were adopted in the peer education program module. This peer educator program is focused on creating an effective network of volunteers who are compassionately engaged with their direct environment mainly their school peers. This process is guided by professional psychologists and trained adult volunteers. Throughout the year, peer educators come together weekly under the guidance of adult volunteers in their own school to learn, openly share, and support each other. Key elements include: (1) theoretical framework for peer educator program—Human capacity for response and Community life competence program, (2) Role of a Peer Educator, (3) Self-Care (Mindfulness and EFT), (4) Active Listening, (5) Understanding Suicide, and (6) Ways to Respond. | Aim: to explore the impact of peer educator programs on the social, emotional, cognitive, behavioural & attitudinal changes among voluntary peer educators in the context of suicide prevention in India. The authors were interested in understanding the changes participants underwent. Or the recruitment of PE volunteers, information was sent out to students and parents, inviting them to a presentation, which was held in the schools. Letters of consent were obtained from each participating PE volunteer & their parents.  Open & axial coding approach was used to analyse data collected from focus groups, one on one interviews & survey. | Themes which are related to the expected outcomes of the program, also known as direct effects & themes which were less anticipated, or indirect effects | Personal development from engaging with the intervention  Increased sense of agency from engaging in PSSP  The intervention providing a space for participants to connect, share experiences and support one another | |  |
| *Note:* ACT= Acknowledge, Care & Tell. CBT= Cognitive-Behavioural Therapy. CBTSI=Cognitive Behavioural Therapy Skills Intervention. DBT=Dialectical Behaviour Therapy*.*  EFT=Emotional Freedom Technique. PAX=Good Behaviour Game. PE = Peer Education*.* SALT= Support, Appreciate, Listen & Team. SEHER=Strengthening Evidence based on  scHool-based intErventions for pRomoting adolescent health programme. SOS=Signs of Suicide. TeenMHFA*=*Teen Mental Health First Aid. YAM=Youth Aware of Mental Health Programme.  ^a^=Number of participants who took part in the qualitative data collection process. ^b^=Reported for overall sample. ^c^=Gender not reported in the study.  **References** | | | | | | | | |

Braun, M., Till, B., Pirkis, J., & Niederkrotenthaler, T. (2021). Suicide prevention videos developed by and for adolescents: A qualitative study [Article]. *Crisis, 42*(2), 114-120. <https://doi.org/10.1027/0227-5910/a000696>

Calear, A. L., Morse, A. R., Batterham, P. J., Forbes, O., & Banfield, M. (2021). Silence is Deadly: A controlled trial of a public health intervention to promote help-seeking in adolescent males. *Suicide and Life-Threatening Behavior, 51*(2), 274-288. <https://doi.org/10.1111/sltb.12703>

Chaniang, S., Fongkaew, W., Stone, T. E., Sethabouppha, H., & Lirtmunlikaporn, S. (2019). Development and evaluation of a suicide prevention program for secondary school students. *Pacific Rim International Journal of Nursing Research, 23*(3), 201-213. <https://he02.tci-thaijo.org/index.php/PRIJNR/article/view/144034>

Chartier, M. J., Phanlouvong, A., Weenusk, J., McCulloch, S., Ly, G., Boyd, L., Murdock, N., Turner, F., Martinson, A., Munro, G., & Sareen, J. (2022). Evaluating the strengths and challenges of PAX dream makers approach to mental health promotion: perspectives of youth and community members in indigenous communities in Manitoba, Canada. *International Journal Of Circumpolar Health, 81*(1). <https://doi.org/10.1080/22423982.2022.2089378>

Hudson, M. M. (2016). *Outcomes of a school-based suicide prevention program for middle school students* (Publication Number 10159502) [Doctoral dissertation, William James College]. <https://www.proquest.com/openview/426c0061f1754db49d3df1a701fb61e4/1?pq-origsite=gscholar&cbl=18750>

Johnson, C. L., Hart, L. M., Rossetto, A., Morgan, A. J., & Jorm, A. F. (2021). Lessons learnt from the field: a qualitative evaluation of adolescent experiences of a universal mental health education program. *Health Education Research, 36*(1), 126-139. <https://doi.org/10.1093/her/cyaa050>

Klim-Conforti, P., Levitt, A. J., Cheung, A. H., Loureiro, R., Fefergrad, M., Schaffer, A., Niederkrotenthaler, T., Sinyor, M., & Zaheer, J. (2022). Youth voices and experiences regarding a school-based cognitive behavioral therapy skills intervention: Lessons for future engagement and adaptation. *BMC Public Health, 22*(1), 1709. <https://doi.org/10.1186/s12889-022-14058-z>

Labouliere, C. D., Tarquini, S. J., Totura, C. M. W., Kutash, K., & Karver, M. S. (2015). Revisiting the concept of knowledge: how much is learned by students participating in suicide prevention gatekeeper training? *Crisis, 36*(4), 274-280. <https://doi.org/10.1027/0227-5910/a000323>

Mirick, R. G., & McCauley, J. (2022). Virtual SOS signs of suicide: Student perspectives and acceptability. *School Psychology Review*, 1-8. <https://doi.org/10.1080/2372966X.2022.2134975>

Ohlmann, C., Kwee, J., & Lees, R. (2014). Listening for the voices of resilience: A group of adolescents’ experiences with a suicide prevention education program. *International Journal of Child, Youth and Family Studies, 5*(1), 24-46. <https://doi.org/10.18357/ijcyfs.ohlmannc.512014>

Quinn, P. (2019). *Building resilience, building the future: evaluating a school-based resilience building programme for second year students* [Doctoral dissertation, University of Limerick]. <https://hdl.handle.net/10344/8580>

Robertson, S. (2008). *Evaluation of the lifesavers high school, peer-support, suicide and crisis-prevention training program* (Publication Number 3383546) [Doctoral dissertation, Southern Illinois University Carbondale ]. psyh. <https://www.proquest.com/openview/69befa73829807443a9e32243b320d9a/1?pq-origsite=gscholar&cbl=18750>

Shinde, S., Pereira, B., Khandeparkar, P., Sharma, A., Patton, G., Ross, D. A., Weiss, H. A., & Patel, V. (2017). The development and pilot testing of a multicomponent health promotion intervention (SEHER) for secondary schools in Bihar, India. *Global Health Action, 10*(1), 1385284. <https://doi.org/10.1080/16549716.2017.1385284>

Wasserman, C., Postuvan, V., Herta, D., Iosue, M., Värnik, P., & Carli, V. (2018). Interactions between youth and mental health professionals: The Youth Aware of Mental health (YAM) program experience. *PLoS One, 13*(2), e0191843. <https://doi.org/10.1371/journal.pone.0191843>

Wexler, L., Poudel-Tandukar, K., Rataj, S., Trout, L., Poudel, K. C., Woods, M., & Chachamovich, E. (2017, 2017/06/01). Preliminary Evaluation of a School-Based Youth Leadership and Prevention Program in Rural Alaska Native Communities. *School Mental Health, 9*(2), 172-183. <https://doi.org/10.1007/s12310-016-9203-2>

Zachariah, B., de Wit, E. E., Bahirat, J. D., Bunders-Aelen, J. F. G., & Regeer, B. J. (2018). What is in it for them? Understanding the impact of a 'Support, Appreciate, Listen Team' (SALT)-based suicide prevention peer education program on peer educators. *School Mental Health, 10*(4), 462-476. <https://doi.org/10.1007/s12310-018-9264-5>
