# Supplementary material for: Review: Adolescents' perspectives on and experiences with post‐primary school‐based suicide prevention as end‐users, co‐creators and peer helpers – a systematic review meta‐ethnography
Source: Child Adolesc Ment Health. 2025 Nov 6;31(3):217–28. doi: 10.1111/camh.70043 (PMC13474300; doi:10.1111/camh.70043)
Supplement: Supplementary file 2 [file CAMH-31-217-s002.docx]

**Supporting Information**

**A meta-ethnography of adolescents’ perspectives on and experiences with post-primary school-based suicide prevention as end-users, co-creators, and peer helpers**

Eibhlin H. Walsh,^1,2,^ Laura Hemming,^3^ Matthew P. Herring,^4,5,^ Jennifer McMahon^1,2^

^1^School, Child & Youth (SCY) Mental Health and Wellbeing Research Lab, Health Research Institute, University of Limerick, Limerick, Ireland

^2^Department of Psychology, University of Limerick, Limerick, Ireland.

^3^Violet Vines Marshman Centre, La Trobe Rural Health School, Melbourne, Australia

^4^Physical Activity *for* Health Cluster, Health Research Institute, University of Limerick, Limerick, Ireland	^5^Department of Physical Education and Sports Sciences, University of Limerick, Limerick, Ireland

**Supplementary Table S1**

*Amendments to Protocol for the Meta-ethnography*

| **Protocol amendments** | **Rationale for amendments** |
| --- | --- |
| **Analysis of some first-order constructs without supporting second-order constructs.** | First-order constructs were mostly analysed alongside their second-order constructs, however some first order constructs were reported in studies without corresponding second order constructs. These first order constructs were nonetheless included given the central focus of youth voice in our meta-ethnography. |
| **Inclusion of studies which partially lacked conceptual depth.** | We retained studies for synthesis which included some conceptual depth and instead excluded codes which lacked conceptual depth, in order to retain as much relevant data as possible. Furthermore, this practice is suitable to our meta-ethnography given that we did not include a particularly large number of studies (<40 studies is considered typical). Moreover, including studies which contribute minimally to the synthesis does not distort the interpretative findings given that the power of the ideas is important in qualitative synthesis (Campbell et al., 2011). Toye et al. (2014) similarly did not exclude studies in entirety that included ‘untranslatable’ concepts. |
| **Lack of reporting of school demographics** | Although school demographics were extracted school characteristics are not included in the data extraction table due to both lack of reporting and consistency of reporting |

**References**

Campbell, R., Pound, P., Morgan, M., Daker-White, G., Britten, N., Pill, R., Yardley, L., Pope, C., & Donovan, J. (2011). Evaluating meta ethnography: systematic analysis and synthesis of qualitative research. *Health Technology Assessment*. <https://doi.org/10.3310/hta15430>

Toye, F., Seers, K., Allcock, N., Briggs, M., Carr, E., & Barker, K. (2014). Meta-ethnography 25 years on: challenges and insights for synthesising a large number of qualitative studies. *BMC medical research methodology, 14*(1), 80. <https://doi.org/10.1186/1471-2288-14-80>

| **Supplementary Table S3**  *CASP Checklist Scores for Retained Meta-ethnography Studies* | | | | | | | | | | | | | | |
| --- | --- | --- | --- | --- | --- | --- | --- | --- | --- | --- | --- | --- | --- | --- |
| **Authors** | **Section A: Are the results of the study valid?** | | | | | | **Section B: What are the results?** | | | | **Section C: Will the results help locally?** | | **Additional Question:**  **Are the interventions of interest clearly described?”** |  |
|  | Q1 | Q2 | Q3 | Q4 | Q5 | Q6 | | Q7 | Q8 | Q9 | | Q10 | Q11 |  |
| **Braun et al. (2021)** | Yes | Yes | Yes | Yes | Yes | Yes | | Yes | Yes | Yes | | Yes | Yes |  |
| **Calear et al. (2021)** | Yes | Yes | Yes | Yes | Yes | Can’t tell | | Yes | Yes | No | | Yes | Yes |  |
| **Chaniang et al. (2019)** | Yes | Yes | Yes | Yes | No | No | | Yes | Yes | Yes | | Yes | Yes |  |
| **Chartier et al. (2022)** | Yes | Yes | Yes | Yes | Yes | Yes | | Yes | Yes | Yes | | Yes | Yes |  |
| **Hudson (2016)** | Yes | Yes | Yes | Yes | Yes | Yes | | Yes | Yes | Yes | | Yes | Yes |  |
| **Labouliere et al. (2015)** | Yes | Yes | Yes | Yes | Yes | No | | Can’t tell | Yes | Yes | | Yes | Yes |  |
| **Johnson et al. (2021)** | Yes | Yes | Yes | Yes | Yes | Yes | | Yes | Yes | Yes | | Yes | Yes |  |
| **Klim-Conforti et al. (2022)** | Yes | Yes | Yes | Yes | Yes | Yes | | Yes | Yes | Yes | | Yes | Yes |  |
| **Mirick and McCauley (2022)** | Yes | Yes | Yes | Yes | Yes | No | | Yes | Yes | Yes | | Yes | Yes |  |
| **Ohlmann et al. (2014)** | Yes | Yes | Yes | Yes | Yes | Yes | | Yes | Yes | Yes | | Yes | Yes |  |
| **Quinn (2019)** | Yes | Yes | Yes | Yes | Yes | Yes | | Yes | Yes | Yes | | Yes | Yes |  |
| **Robertson (2008)** | Yes | Yes | Yes | Yes | Yes | No | | Yes | Yes | Yes | | Yes | Yes |  |
| **Shinde et al. (2017)** | Yes | Yes | Yes | Yes | Yes | No | | Yes | Yes | Yes | | Yes | Yes |  |
| **Wasserman et al. (2018)** | Yes | Yes | Yes | Yes | Yes | Yes | | Yes | Yes | Yes | | Yes | Yes |  |
| **Wexler et al. (2017)** | Yes | Yes | Yes | Yes | Yes | No | | Can’t tell | Yes | Yes | | Yes | Yes |  |
| **Zachariah et al. (2018)** | Yes | Yes | Yes | Yes | Yes | No | | Yes | Yes | Yes | | Yes | Yes |  |
